# Case report of stiff - person syndrome and literature review

**DOI:** 10.1016/j.ibneur.2025.12.006

**Published:** 2026-01-03

**Authors:** Qiongfang Zhang, MengJie Xia, Dan Gui, Jia Wei, Yongfeng Liu, Yanhua Gou

**Affiliations:** a Department of Electromyography, Shenzhen Traditional Chinese Medicine Hospital, Shenzhen, China; b Department of Clinical Neurophysiology, Ruijin Hospital Affiliated to Shanghai Jiao Tong University School of Medicine, Shanghai, China; c Department of Neurology, Shenzhen Traditional Chinese Medicine Hospital, Shenzhen, China; d Department of Acupuncture and Moxibustion, Shenzhen Traditional Chinese Medicine Hospital, Shenzhen, China

**Keywords:** Stiff Person Syndrome (SPS), Needle Electromyography (EMG), Diazepam, Anti-GAD65 Antibody, Case Report

## Abstract

**Objective:**

Currently, there is no objective evaluation method to measure muscle tension and therapeutic effects in patients with stiff person syndrome (SPS). We aimed to investigate objective evaluation criteria for diagnosis and treatment. We used needle electromyography (EMG) to record muscle electrical signals before and after the diazepam drug trial to diagnose a patient with SPS.

**Method:**

We present a patient who was misdiagnosed as having an "anxiety state" for more than a decade because of recurrent tension, worry, and intermittent fear, resulting in generalized muscle stiffness and even falls. Conventional needle EMG was performed on the limbs and axial muscles. The changes in muscle electrical signals before and after diazepam drug trials were recorded.

**Results:**

EMG revealed a significant increase in spontaneous motor unit potentials in the patient's limbs and axial muscles at rest. After intravenous injection of diazepam, the spontaneous motor unit gradually reduced and ultimately disappeared. Based on these results, anti-antibody testing for SPS was performed one week later. The results revealed a high titer of anti-glutamic acid decarboxylase 65 antibodies (GAD65-Abs) in serum, while anti-amphiphysin antibodies were negative. The diagnosis was confirmed as SPS.

**Conclusion:**

Needle EMG is a convenient tool that can provide diagnostic direction for SPS before genetic testing, enabling clinicians to select specific genes for testing and thereby shortening the time required for clinical examination and diagnosis for patients. Additionally, it can be used to verify the efficacy of experimental medications during testing.

## 1 Introduction

Stiff Person Syndrome (SPS) is a rare neurological disorder first described and named by Moersch and Woltman in 1956 (Moersch and Woltman, 1956), characterized primarily by muscle stiffness and spasms that frequently involve the trunk and proximal limb muscles. While the pathogenesis of SPS has not yet been fully elucidated, subsequent research has advanced our understanding of its biological and clinical features. Solimena et al., 1988, Lohmann et al., 2000 identified SPS as a distinct autoimmune disease, and over 70 % of SPS cases are associated with the presence of serum anti-glutamic acid decarboxylase (GAD) antibodies (anti-GAD antibodies) (Lee et al., 2019)—a marker first proposed for inclusion in SPS diagnostic criteria by Gordon et al. (1967).

Epidemiologically, SPS exhibits a very low overall prevalence of approximately 1–2 cases per million individuals (Dalakas, 2022, Baizabal-Carvallo and Jankovic, 2015), with a notable predilection for postmenopausal women. A nationwide survey conducted in Japan identified 30 GAD65-positive SPS cases, leading to an estimated total of 140 GAD65-positive SPS patients and a prevalence rate of 0.11 per 100,000 population. In this cohort, the median age of onset was 51 years, 76 % of patients were female, and 70 % were classified as having classic SPS (Barker et al., 1998). Clinically, the initial symptoms of SPS typically manifest as muscle stiffness in the axial and proximal regions, which gradually progresses to the distal limbs (Buechner et al., 2015). These symptoms are frequently exacerbated by external or internal stimuli, including noise, touch, sudden movement, emotional distress, and stress (Baizabal-Carvallo and Jankovic, 2015, Meinck and Thompson, 2002, Egwuonwu and Chedebeau, 2010), ultimately resulting in difficulties with ambulation and gait imbalance, and an increased risk of falls and fractures (Buechner et al., 2015).

The pathogenic link between GAD65 and SPS is central to understanding the disease. Gamma-aminobutyric acid (GABA)—an inhibitory neurotransmitter in the central nervous system (Gao, 2022, Saengboonmee et al., 2023, Yamazaki et al., 2020)—is synthesized via a reaction catalyzed by GAD (glutamic acid decarboxylase), a rate-limiting enzyme also present in pancreatic β cells (Baizabal-Carvallo and Jankovic, 2015, Bhatti and Gazali, 2015). The GAD65 protein, encoded by the GAD2 gene located on chromosome 10 p11.23, specifically catalyzes on-demand GABA production in synaptic vesicles (Zeng et al., 2023, Schieweck and Kiebler, 2019). Anti-GAD antibodies, however, disrupt this process: their presence impairs the function of both neurons and pancreatic β cells (Solimena et al., 1988), leading to deficiencies in GABA synthesis (Solimena et al., 1988, Lohmann et al., 2000) and an imbalance in inhibitory neurotransmission. This imbalance induces excessive neuronal excitation, which manifests as muscle spasms in SPS patients (Hermans et al., 2018). Importantly, SPS shares autoimmune associations with other conditions, particularly type 1 diabetes mellitus (T1DM): GAD65 autoantibodies are detected in 80 % of newly diagnosed T1DM patients (Patel et al., 2020, Balint and Bhatia, 2016), though antibody titers in SPS are significantly higher (Bai et al., 2022, Trier et al., 2022). A retrospective analysis of 121 patients with SPS spectrum disorder further confirmed this link: 41.3 % (50/121) had classic SPS, 65.4 % (34/52) of whom had concurrent systemic autoimmune diseases, and 86.5 % (45/52) exhibited elevated GAD65 antibody levels (Martinez-Hernandez et al., 2016).

In clinical practice, diagnostic and therapeutic standards for SPS have evolved over time. In 2009, researchers established formal clinical criteria for classic SPS, which include: (1) progressive muscle stiffness and spasms affecting axial muscles, causing significant gait impairment; (2) symptom triggering by sudden movements, noise, or emotional distress; (3) clinical and electromyographic (EMG) evidence of sustained motor unit activity in both agonist and antagonist muscles; (4) exclusion of other neurological diseases causing stiffness; (5) detection of GAD65 autoantibodies via immunocytochemistry, radioimmunoassay, or Western blotting; and (6) a positive response to diazepam treatment (Dalakas, 2022). Diazepam—a benzodiazepine with muscle relaxant and anti-anxiety properties—acts as a GABA-A receptor agonist (Kitamura et al., 2019), and its therapeutic efficacy for SPS, documented by Olafson et al (Olafson et al., 1964). based on data from the Mayo Clinic Neurology Practice, led to its inclusion in diagnostic criteria. However, the duration and efficacy of symptom improvement (e.g., for muscle stiffness and spasms) vary significantly among individuals (Patel et al., 2020, Zdziarski, 2015).

Currently, there is a paucity of clinical trials investigating EMG for SPS, and subjective evaluations (e.g., post-administration physical examinations) fail to meet objective diagnostic and therapeutic assessment standards. EMG thus holds substantial clinical value for diagnosing SPS and objectively evaluating experimental treatments. Against this backdrop, we present a rare case of SPS in a middle-aged Chinese female with comorbid T1DM, thyroid cancer, and pulmonary tuberculosis. Using EMG technology, we recorded the patient’s spontaneous muscle motor potential activity and observed the gradual reduction and disappearance of spontaneous motor units following experimental intravenous diazepam administration. Our findings aim to establish objective, precise criteria for the experimental diagnosis and treatment evaluation of SPS.

## 2 Case introduction

The patient is a 54-year-old middle-aged woman with a history of T1DM, post-thyroid adenocarcinoma surgery, hypothyroidism, and pulmonary tuberculosis.

Medical history review: A decade ago, the patient had a car accident while carrying a large amount of cash to the bank for work. She subsequently began to experience tension, fear, and muscle stiffness in her body as a result of theft at home and other factors. She would experience tension, fear, or even collapse and sustain an injury when she heard sudden noises. Since 2010, she has been treated in hospitals in Guangzhou, Hong Kong, and Shenzhen, diagnosed with "anxiety disorder," and prescribed medications, including duloxetine 120 mg qd, clonazepam, and alprazolam. However, the symptoms did not exhibit a significant improvement and tended to recur. One month ago, the patient sought treatment for traditional Chinese medicine at the Department of Neurological and Psychiatric Diseases at Shenzhen Traditional Chinese Medicine Hospital's outpatient clinic. She was prescribed "Shugan Jieyu Capsules" in combination with oral Chinese medicine; however, the effect was unsatisfactory. On July 14, 2025, the patient was admitted to the hospital with the primary complaint of over 10 years of intermittent tension, worry, and fear, accompanied by progressive limb stiffness for 3 years, and diagnosed with depression and an anxiety state.

The patient appeared generally alert at the time of admission; however, she exhibited anxiety and concern, particularly in crowded environments. She was sensitive, slightly low in mood and interest, occasionally experiencing palpitations and irritability, occasional self-blame, and a tendency to cry easily. When she was anxious, she experienced profuse sweating and complained of lower back pain. There was no lymphadenopathy, edema, chest tightness or pain, jaundice, or other symptoms. Appetite and sleep were normal, with frequent dreams. Bowel movements occurred 1 −2 times a day, and urination was normal. There was no significant change in weight recently. Family history: Father has a history of hyperthyroidism and diabetes, while mother and children are healthy. Neurological Physical Examination: Meningeal irritation signs were negative (-); muscle strength of all four limbs was Grade Ⅴ; muscle tone of all four limbs was slightly increased, with a significant increase during episodes; tendon reflexes of all four limbs were elicited symmetrically; ataxia was negative (-), and ataxia-related tests such as Romberg's sign, finger-to-nose test, and heel-to-knee-to-shin test all showed normal results; pathological reflexes of both lower limbs were negative (-), and reflexes such as Babinski sign and Chaddock sign were not elicited; during episodes, the patient presented with a "wooden man gait", characterized by stiff limbs, reduced movement amplitude while walking, and a posture similar to a sculpture; cranial nerve examination results were negative (-), with normal visual acuity, visual field, eye movement, facial sensation and movement, hearing, and swallowing reflex.

After admission, the patient underwent examinations, including peripheral nerve conduction and needle EMG due to muscle stiffness in the limbs and trunk accompanied by pain symptoms. This study was approved by the institutional review board, and written informed consent was obtained from the patient.

## 3 Results

### 3.1 Laboratory tests

Laboratory test results indicated fasting blood glucose of 13.75 mmol/L ↑ (3.96–6.12 mmol/L), 2-h postprandial blood glucose of 22.45 mmol/L ↑ (3.96–7.80 mmol/L), and hemoglobin A1c of

9.4 % ↑ (4–6 %). Thyroid function testing revealed triiodothyronine of 0.93 nmol/L ↓ (1.01–2.48nmol/L), thyroid peroxidase antibody at 90.96 IU/mL ↑ (0.00–4.00IU/mL), thyroglobulin antibody > 991.01 IU/mL ↑ (0.0–9.0IU/mL), and thyroglobulin at 0.1 µg/L ↓ (1.6–50.0ug/L). The blood lipid profile indicated a high-density lipoprotein cholesterol level of 2.36 mmol/L ↑ (0.90–1.55 mmol/L). Biochemical analysis revealed a total protein concentration of 61.7 g/L ↓ (65.0–85.0 g/L), albumin of 39.8 g/L ↓ (40.0–55.0 g/L), and alpha-hydroxybutyrate dehydrogenase activity of 189 U/L ↑ (72–182 U/L).

### 3.2 Imaging examination results

**Fig. 1:**
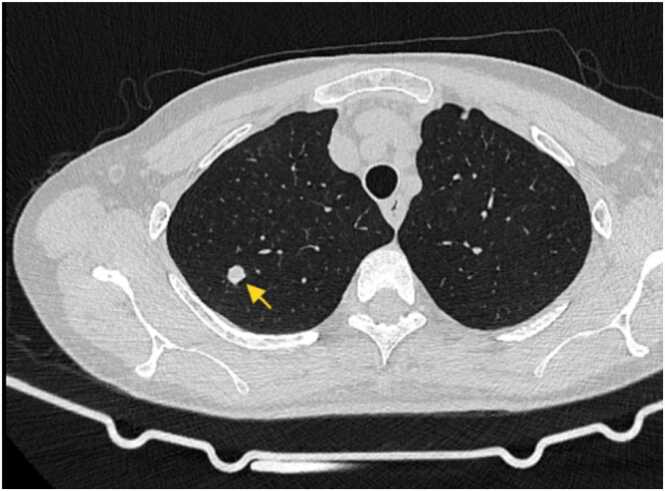
Axial chest CT (lung window): Bilateral secondary pulmonary tuberculosis (arrow), multiple old rib fractures, and aortic wall calcification.

**Fig. 2:**
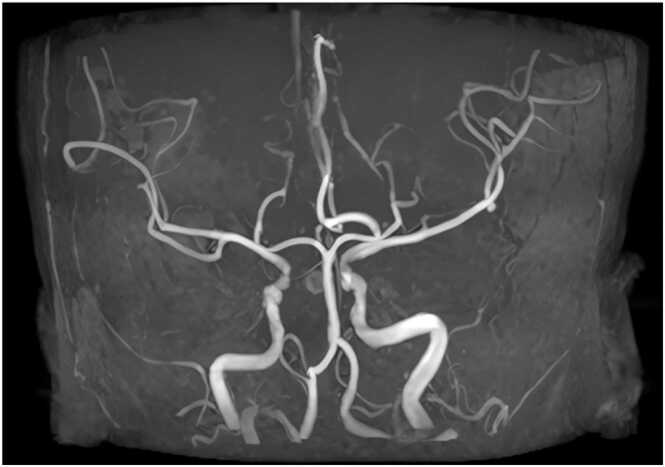
Cerebral MRA (MIP reconstruction): Anatomical variation of the right anterior cerebral artery (hyperintensity); no vascular stenosis/occlusion.

**Fig. 3:**
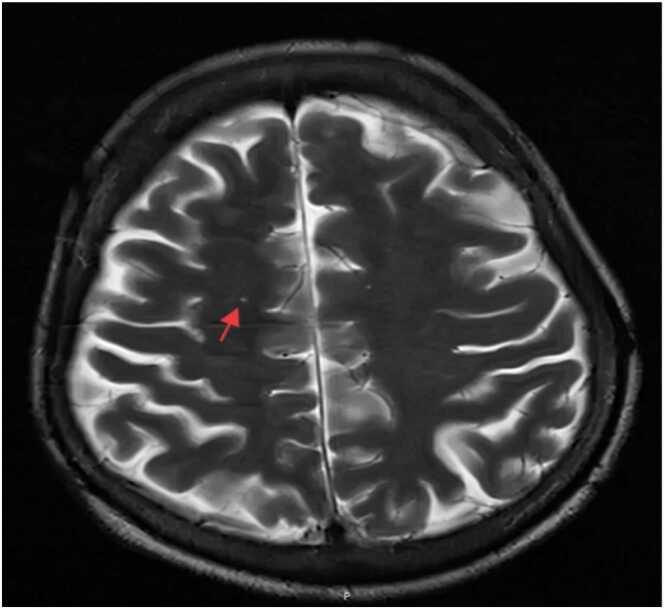
Axial brain MRI (FLAIR sequence): Periventricular white matter ischemia (arrow); no acute cerebral infarction/hemorrhage.

**Fig. 4:**
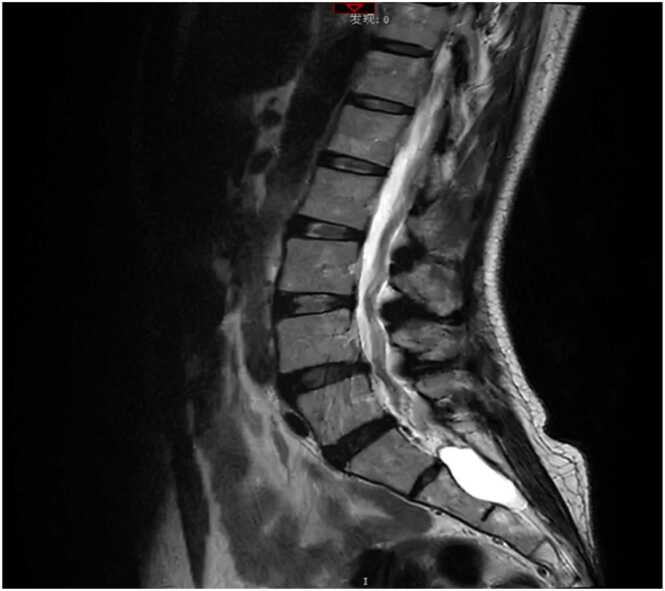
Sagittal lumbar spine T2-weighted MRI: L3/4-L5/S1 disc bulging, lumbar degenerative changes, and a sacral canal cyst.

Chest computed tomography (Fig. 1) on May 7, 2025, revealed secondary pulmonary tuberculosis in both lungs, multiple old rib fractures on both sides, and aortic wall calcification. On July 17, 2025, brain and cerebral artery magnetic resonance angiography (MRA) (Fig. 2) and MRA plain scan (Fig. 3) exhibited high signal intensity in white matter (Fazekas I grade). The A1 segment of the right anterior cerebral artery was absent, indicating an anatomical variation. Cerebral artery hardening was observed, and mild inflammation was present in both paranasal sinuses. Additionally, lumbosacral magnetic resonance imaging (Fig. 4) revealed disc bulging at L3/4, L4/5, and L5/S1, degenerative changes in the lumbar spine, and the presence of a sacral canal cyst.

### 3.3 Electrophysiological test results

**(1)Electrophysiological testing method**

The examination was conducted in a calm environment at a room temperature of 25 −30 °C using the Natus Neurolog EMG evoked potential instrument (Natus Neurolog, Inc., USA). The hands were heated as required to maintain palm temperature at 32 °C or above. The subject was positioned in a supine position with relaxed muscles, and a bilateral examination was performed for comparative observation.

(2) Operating method

**① Nerve conduction studies**

The methods for motor conduction, sensory conduction, and F-wave studies are based on the detection methods and diagnostic criteria from "EMG and neuromuscular disorders e-book: clinical-electrophysiologic-ultrasound correlations" (Preston and Shapiro, 2020). The results were all normal without any significant abnormalities.

**② EMG testing**

**Fig. 5:**
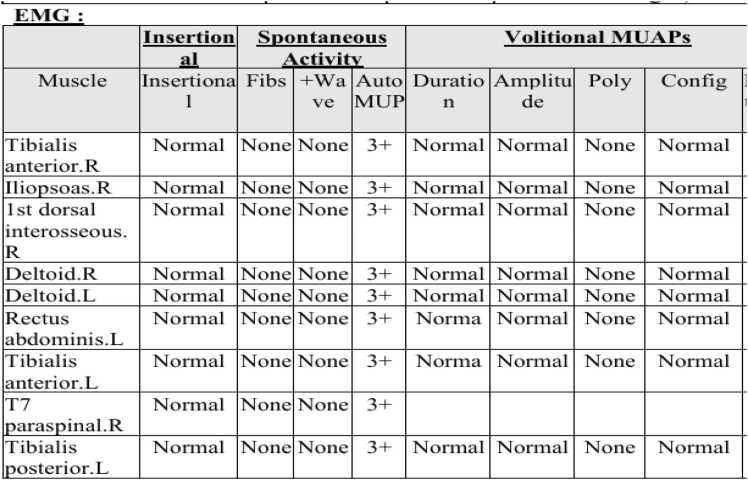
Electromyography (EMG) findings of motor unit action potentials (MUAPs) across muscle groups. Concentric needle electrodes were used to sequentially test proximal/distal limb, paraspinal, sternocleidomastoid, and rectus abdominis muscles. The table summarizes MUAP characteristics (insertion/spontaneous activity, volitional MUAP duration/amplitude/polyphasia/configuration) recorded in resting, light,and heavy contraction states. Abbreviations: MUAP = motor unit action potential; R = right; L = left.

Using concentric needle electrodes, the muscles of the proximal and distal limbs, as well as the paraspinal, sternocleidomastoid, and rectus abdominis muscles, were tested sequentially (Fig. 5). The morphology and changes of the motor unit potential (MUP) in the resting state, light contraction, and heavy contraction states were recorded.

**③ Diazepam Test:**

Diazepam Injection: 10 mg, iv.

**Fig. 6:**
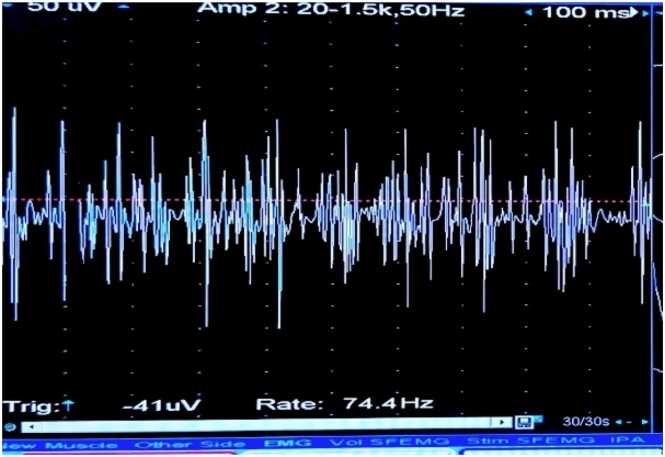
Before diazepam injection (17:37).

**Fig. 7:**
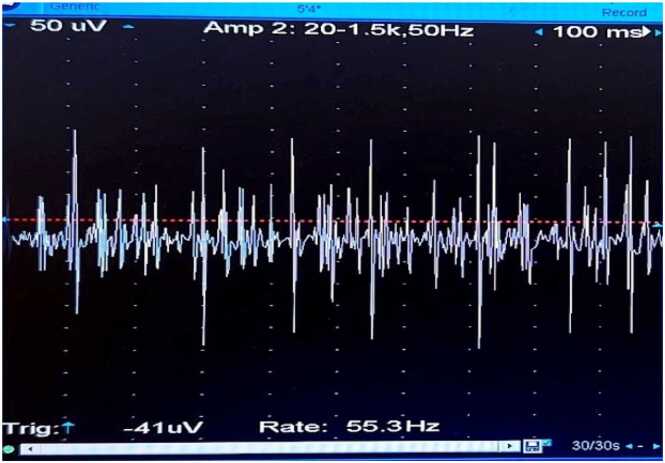
After diazepam injection at 0 min (17:41:50).

**Fig. 8:**
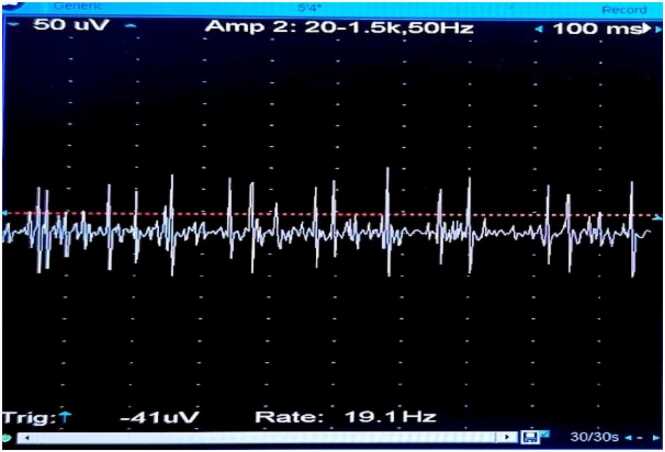
1 min after the diazepam injection (17:42:50).

**Fig. 9:**
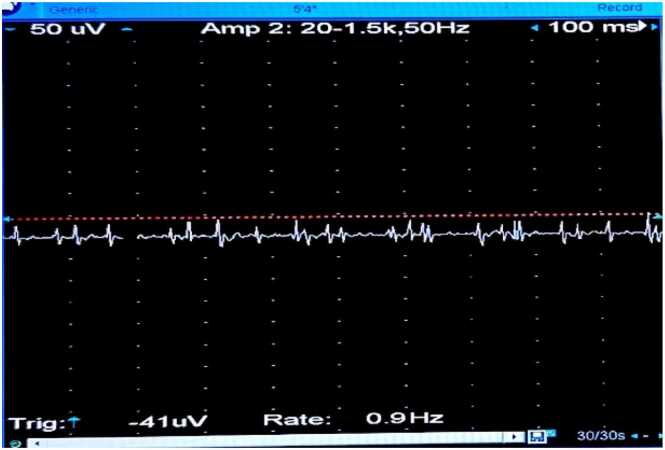
2 min after diazepam injection (17:43:50).

**Fig. 10:**
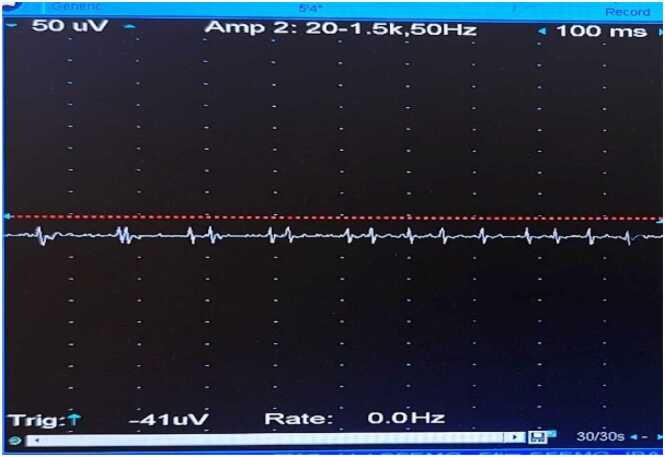
3 min after diazepam injection (17:44:50).

**Fig. 11:**
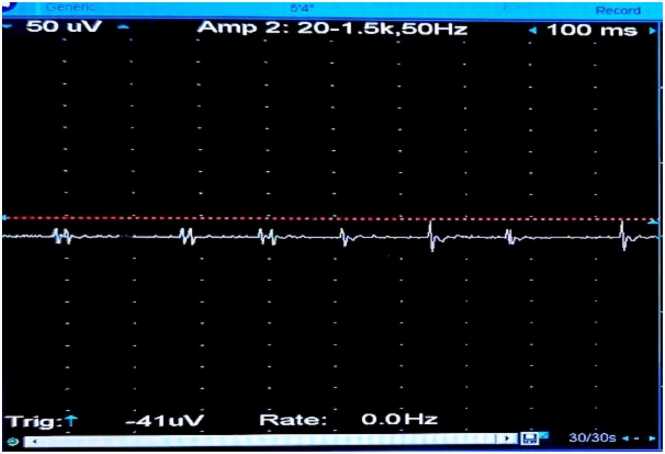
4 min after diazepam injection (17:45:50).

**Fig. 12:**
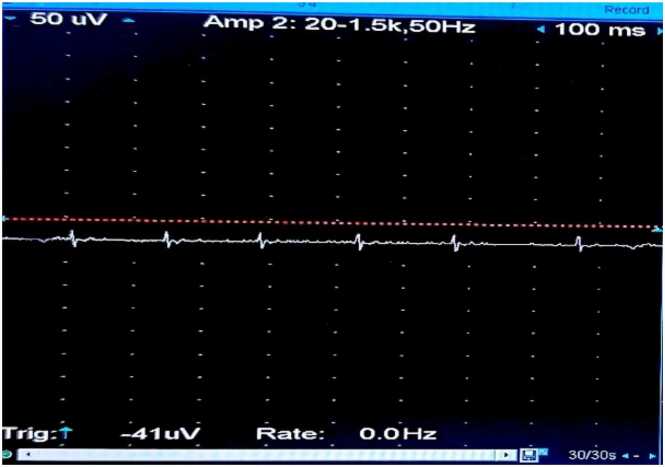
5 min after diazepam injection (17:46:50).

**Fig. 13:**
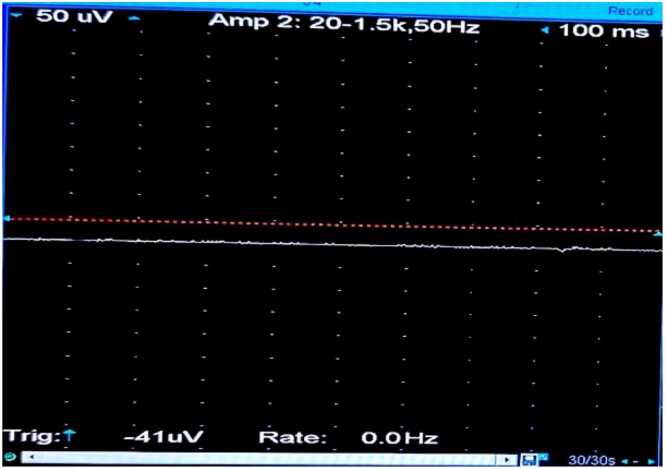
6 min after diazepam injection (17:47:50).

The concentric circle needles were inserted into the deltoid muscle of the right upper arm. The MUP status was recorded before the diazepam bolus (Fig. 6), after the bolus at 0 min (Fig. 7), 1 min (Fig. 8, Fig. 2 min (Fig. 9, Fig. 3 min (Fig. 10, Fig. 4 min (Fig. 11, Fig. 5 min (Fig. 12). As time progresses, the resting spontaneous action potential gradually decreases in amplitude. At 6 min after the diazepam injection (Fig. 13), the automatic MUP completely disappears. The results are as follows:

### 3.4 Genetic test (2025.07.23)

On July 23, 2025, an anti-GAD65 (G065) antibody test was performed, which exhibited positive results, with the detected antibody concentration level being 1:320.

## 4 Discussion

### 4.1 The core role of EMG in SPS diagnosis

Electromyography (EMG) is an indispensable tool for SPS diagnosis, as its ability to detect characteristic abnormalities directly aligns with the third criterion of classic SPS (sustained motor unit activity in agonist and antagonist muscles) (Dalakas, 2022). Multiple studies have validated its diagnostic utility: in a case report of a 36-year-old male with an 11-year history of progressive lower limb stiffness and painful spasms, EMG revealed continuous motor unit activity in paravertebral and leg muscles—findings that confirmed a diagnosis of autoimmune SPS (Souissi et al., 2020). Similarly, an observational study of five SPS patients found that all exhibited disease-specific EMG abnormalities, reinforcing EMG’s role in confirming SPS (Kumawat et al., 2024).

Notably, combining EMG with antibody testing significantly enhances diagnostic accuracy. A retrospective case-control study of 154 patients with classic SPS, 45 with SPS-plus, and 66 controls demonstrated a > 90 % correlation between classic SPS and characteristic EMG abnormalities (Roy et al., 2025). This synergy is particularly valuable for early diagnosis: in cases with subtle clinical symptoms or borderline antibody titers, EMG-detected continuous motor unit activity can provide definitive evidence of SPS, enabling timely initiation of targeted treatment.

### 4.2 The EMG diazepam test: a tool for objective therapeutic evaluation

Despite its inclusion in SPS diagnostic criteria, the clinical utility of diazepam is limited by interindividual variability in treatment response (Patel et al., 2020, Zdziarski, 2015). The EMG diazepam test addresses this gap by providing an objective measure of treatment efficacy—yet research on this application remains scarce. Mechanistically, diazepam binds to central nervous system benzodiazepine receptors, enhancing GABA’s affinity for its receptors, promoting GABAergic neurotransmission, and inducing neuronal hyperpolarization via chloride ion influx. This process inhibits neuronal excitability, alleviates muscle stiffness and spasms (Bhatti and Gazali, 2015), and partially reverses the inhibitory neurotransmission imbalance caused by anti-GAD antibodies.

Clinical observations support the test’s value: some SPS patients exhibit reduced EMG-detected muscle activity abnormalities concurrent with symptomatic improvement (e.g., less stiffness or spasms) following diazepam administration (Lin et al., 2021).For clinicians, this objective correlation enables personalized treatment planning: patients with marked reductions in spontaneous motor units on EMG may benefit from long-term diazepam therapy, while those with minimal EMG changes can be considered for alternative treatments (e.g., immunotherapies). In this way, the EMG diazepam test bridges the gap between subjective symptom reporting and objective physiological data, optimizing therapeutic decision-making.

### 4.3 Clinical significance of the reported case

The case presented herein—a middle-aged Chinese female with SPS and comorbid T1DM, thyroid cancer, and pulmonary tuberculosis—is clinically notable for two key reasons. First, it highlights the overlap between SPS and other autoimmune conditions: T1DM, like SPS, is associated with GAD65 autoantibodies (Bai et al., 2022, Trier et al., 2022), and the co-occurrence of these diseases underscores the need for comprehensive autoimmune screening in SPS patients. Second, the case demonstrates the practical value of EMG in real-world clinical settings: pre-diazepam EMG recorded spontaneous motor potentials consistent with SPS, while post-diazepam EMG showed gradual reduction and disappearance of these potentials—objective evidence of diazepam’s efficacy in this patient.

This case also addresses a critical limitation of current SPS care: the reliance on subjective assessments. By linking symptomatic improvement (e.g., reduced spasms) to quantifiable EMG changes, the case validates the EMG diazepam test as a tool for monitoring treatment response in complex patients (e.g., those with multiple comorbidities that may confound clinical evaluations).

### 4.4 Future prospects for EMG in SPS

Electromyography (EMG) harbors substantial untapped potential to advance the diagnosis, prognosis, and management of stiff person syndrome (SPS). First, refined EMG signal analysis—encompassing the quantification of motor unit potential (MUP) morphology, frequency, and amplitude—could facilitate more precise staging of disease severity and real-time monitoring of disease progression. Moreover, integrating EMG with nerve conduction velocity (NCV) testing may further elucidate the extent of peripheral nervous system involvement in SPS, thereby generating a more comprehensive neuromuscular assessment profile.

Second, exploring the utility of EMG in evaluating the efficacy of alternative therapeutic agents for SPS—such as intravenous immunoglobulin (IVIG) and rituximab—will contribute to the establishment of a holistic, objective evaluation system for SPS treatment outcomes. Notably, if focal EMG abnormalities are demonstrated to correlate with regional neuronal dysfunction, targeted drug delivery or neuromodulation strategies could be investigated as promising avenues for precision therapy in SPS.

In conclusion, EMG plays an irreplaceable role in the diagnosis and therapeutic evaluation of SPS, particularly in resource-limited healthcare settings. Resource constraints in such contexts frequently give rise to challenges including subjective assessment bias and inadequate follow-up monitoring; EMG addresses these gaps by providing objective, quantifiable evidence. Specifically, when combined with antibody testing, EMG not only enhances diagnostic accuracy (mitigating the risk of missed diagnoses associated with antibody testing alone) but also enables the documentation of changes in spontaneous motor units during treatment evaluation (e.g., the reduction or disappearance of potentials following intravenous diazepam administration). This capability replaces experience-dependent subjective physical examinations, thus minimizing diagnostic and therapeutic errors stemming from inconsistent assessment criteria. Furthermore, EMG excludes confounding effects of comorbidities (e.g., type 1 diabetes mellitus, thyroid cancer) via objective physiological data, reducing reliance on multidisciplinary collaboration or specialized tests for differential diagnosis.

Collectively, EMG offers practical technical support for the early diagnosis, treatment optimization, and efficacy monitoring of SPS in resource-limited settings, significantly improving the accessibility and standardization of care for this rare neurological disorder.

## Ethical Publication Statement

We confirm that we have read the Journal’s position on issues involved in ethical publication and affirm that this report is consistent with those guidelines.

## Declarations

Ethics approval and consent to participate

As the data in the EMG reports were anonymized and de - identified before being accessed for this study, and the analysis did not involve any direct interaction with patients or potential risks to their privacy, ethical approval was waived in accordance with the ethical review guidelines of Ethics Committee of Shenzhen Traditional Chinese Medicine Hospital.

## Authors ’ contributions

Yongfeng Liu、Yanhua Gou conceived the research plan for this project; Dan Gui、Jia Wei provided patients' clinical medical records, test results, and imaging reports; Qiongfang Zhang conducted electromyography tests on patients; Qiongfang Zhang、Mengjie Xia analyzed the electromyography results; Qiongfang Zhang wrote the manuscript.

## Consent for publication

All authors have read and approved the manuscript submitted.

## Funding

10.13039/501100012151Sanming Project of Medicine in Shenzhen (SZZYSM202311002).

## CRediT authorship contribution statement

**Dan Gui:** Writing – original draft, Resources. **Jia Wei:** Writing – original draft, Resources. **Qiongfang Zhang:** Writing – review & editing, Writing – original draft, Resources, Project administration, Methodology, Funding acquisition, Formal analysis, Data curation, Conceptualization. **Mengjie Xia:** Writing – review & editing, Writing – original draft, Formal analysis. **Yongfeng Liu:** Writing – review & editing, Writing – original draft, Formal analysis, Conceptualization. **Yanhua Gou:** Writing – review & editing, Project administration, Methodology, Conceptualization.

## Declaration of Competing Interest

The authors declare that there are no conflicts of interest that could influence the research outcomes or compromise the integrity of the study.
